# Brace technology thematic series - The Sforzesco and Sibilla braces, and the SPoRT (Symmetric, Patient oriented, Rigid, Three-dimensional, active) concept

**DOI:** 10.1186/1748-7161-6-8

**Published:** 2011-05-09

**Authors:** Stefano Negrini, Gianfranco Marchini, Fabrizio Tessadri

**Affiliations:** 1 ISICO (Italian Scientific Spine Institute), Milan, Italy; 2 Centro Ortopedico Lombardo, Milan, Italy; 3 Orthotecnica, Trento, Italy

## Abstract

**Background:**

Bracing is an effective strategy for scoliosis treatment, but there is no consensus on the best type of brace, nor on the way in which it should act on the spine to achieve good correction. The aim of this paper is to present the family of SPoRT (Symmetric, Patient-oriented, Rigid, Three-dimensional, active) braces: Sforzesco (the first introduced), Sibilla and Lapadula.

**Methods:**

The Sforzesco brace was developed following specific principles of correction. Due to its overall symmetry, the brace provides space over pathological depressions and pushes over elevations. Correction is reached through construction of the envelope, pushes, escapes, stops, and drivers. The real novelty is the drivers, introduced for the first time with the Sforzesco brace; they allow to achieve the main action of the brace: a three-dimensional elongation pushing the spine in a down-up direction.

Brace prescription is made plane by plane: frontal (on the "slopes", another novelty of this concept, i.e. the laterally flexed sections of the spine), horizontal, and sagittal. The brace is built modelling the trunk shape obtained either by a plaster cast mould or by CAD-CAM construction. Brace checking is essential, since SPoRT braces are adjustable and customisable according to each individual curve pattern.

Treatment time and duration is individually tailored (18-23 hours per day until Risser 3, then gradual reduction). SEAS (Scientific Exercises Approach to Scoliosis) exercises are a key factor to achieve success.

**Results:**

The Sforzesco brace has shown to be more effective than the Lyon brace (matched case/control), equally effective as the Risser plaster cast (prospective cohort with retrospective controls), more effective than the Risser cast + Lyon brace in treating curves over 45 degrees Cobb (prospective cohort), and is able to improve aesthetic appearance (prospective cohort).

**Conclusions:**

The SPoRT concept of bracing (three-dimensional elongation pushing in a down-up direction) is different from the other corrective systems: 3-point, traction, postural, and movement-based. The Sforzesco brace, being comparable to casting, may be the best brace for the worst cases.

## Background

Bracing is an effective strategy for scoliosis treatment, even if proof regarding its efficacy is currently still weak [1,2]. Nevertheless, since the efficacy of bracing comes from both good quality construction and good compliance [3], bracing should never be interpreted only in terms of the brace applied, but also in terms of the management of patients [4]. In fact, compliance is a characteristic neither of the treatment only, nor of the patient alone, but of the good interaction between these two factors and an expert treatment team able to reduce the burden of the brace and increase the coping abilities of the patient.

The expert members of the international Society on Scoliosis Orthopaedic and Rehabilitation Treatment (SOSORT) have not been able to reach a consensus on an optimal brace design, nor on the way it should act on the spine to achieve good correction [5]; on the contrary, they have reached consensus on the proper management of patients to achieve good results [4]. Looking at the existing studies performed using the Scoliosis Research Society (SRS) methodological criteria, and dividing them into two groups (one respecting also the SOSORT criteria [6,7], and another not doing so) it appears that the best results are obtained by the first group [8]. So, the currently available international knowledge seems to agree that the type of brace used is less important than the way in which a brace is applied (SOSORT criteria) [4].

Nevertheless, this way of thinking could drive the field to a form of nihilism, where what you do (brace) is less important than how you do it (SOSORT criteria). Consequently, a comparison among the different tools applied by different physicians is mandatory, in order to understand these tools and to be able to separate their different indications. Until now, there have been very few comparison studies on different braces: one RCT [9], and some studies mainly with historical controls [10-16]. A critical assessment of some of these studies is vital, since in certain cases there has been doubt that the authors were experts in the use of the types of braces evaluated in the study. As a consequence, a more sound understanding of the basis behind the use of different braces is required to increase common background knowledge and to finally be able to safely compare the different instruments.

**Figure 1:**
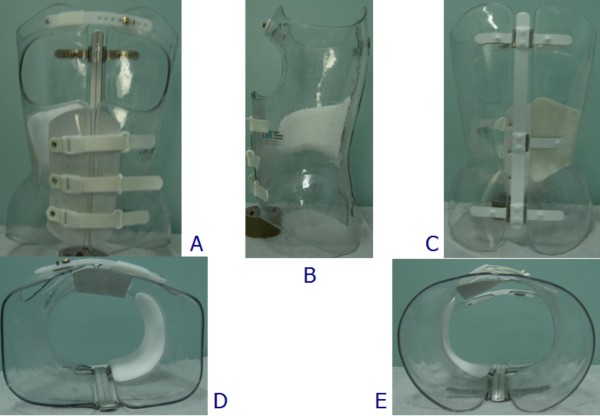
**The Sforzesco brace**. The Sforzesco brace: anterior (A), left (B), posterior (C), top (D), and bottom (E) views.

**Figure 2:**
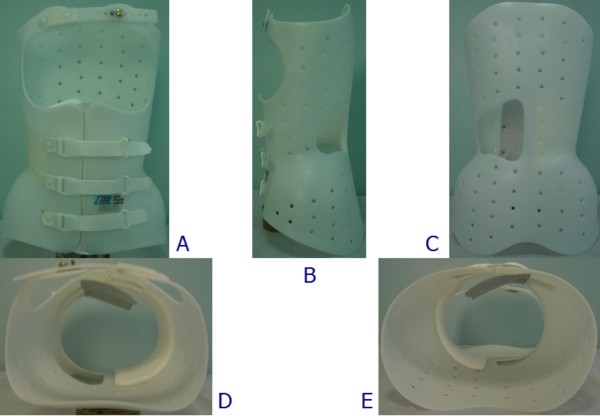
**The Sibilla brace**. The Sibilla brace: anterior (A), left (B), posterior (C), top (D), and bottom (E) views.

**Figure 3:**
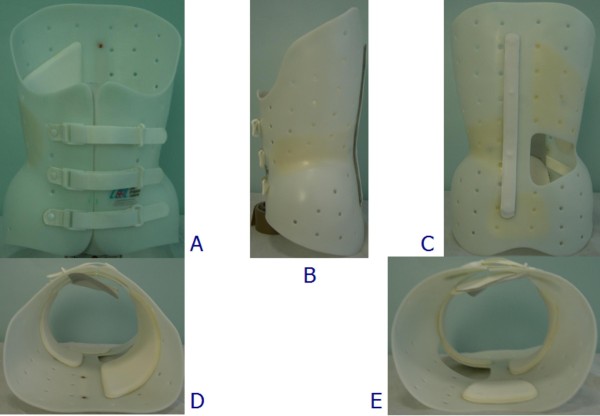
**The Lapadula brace**. The Lapadula brace: anterior (A), left (B), posterior (C), top (D), and bottom (E) views.

The aim of this paper is to present in a journal article format the SPoRT braces (Sforzesco, Figure 1; Sibilla, Figure 2; and Lapadula, Figure 3), which today constitute a family of braces constructed following a single concept of bracing (SPoRT). A complete booklet version of this work can be freely downloaded http://www.isico.it/uk/sforzesco.

## History

**Figure 4:**
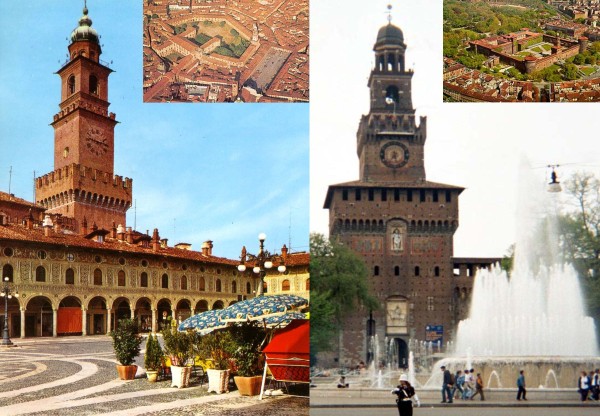
**The Sforzesco brace owns its name to the Sforesco castles of Milan and Vigevano**. The Sforzesco brace was named according to the two main cities of the experience of the main author (SN): Vigevano and Milano, which both have castles named Sforzesco for the Medieval Sforza family.

The Sforzesco brace, named in honour of the Medieval Sforza family (Figure 4), was developed by two of the authors (SN and GM) in the autumn of 2004 while searching for a way to avoid casting for the worst patients Subsequently, the SPoRT (Symmetric, Patient-oriented, Rigid, Three-dimensional, active) concept of bracing was developed [10,17,18]. which also included the previously existing Lapadula and Sibilla brace designs [19,20].

**Figure 5:**
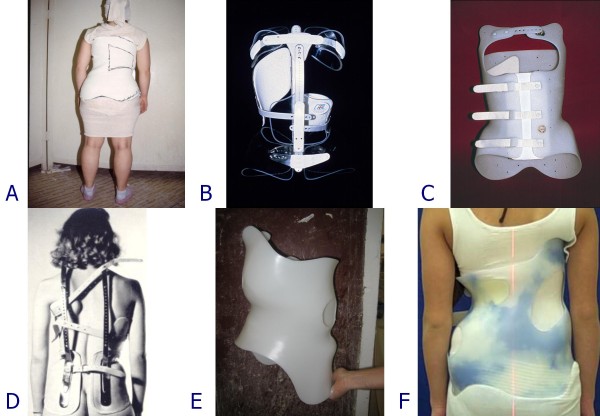
**Braces at the base of the SPoRT concept development**. The concept of SPoRT bracing was developed from the following braces: Risser cast (A), Lyon (B), Sibilla (C) and Milwaukee (D). The last changes made to the SPoRT braces also allowed us to consider among their ancestors the last Cheneau brace (E) and the Rigo Cheneau brace (F).

In the development and construction of the Sforzesco brace, it is possible to recognise elements of various previously-developed braces: Risser cast [20-22] (Figure 5A), Lyon [23] (Figure 5B), Sibilla [19,20] (Figure 5C), and Milwaukee [24,25] (Figure 5D) braces. After the first development, "contaminations" with braces from expert builders from all over the world (i.e. changes made looking at other concepts) was achieved, including now elements from the Cheneau (Figure 5E) [26-29] and Rigo Cheneau System (RCS) (Figure 5F) [26,28,30] braces.

## Theoretical principles

**Figure 6:**
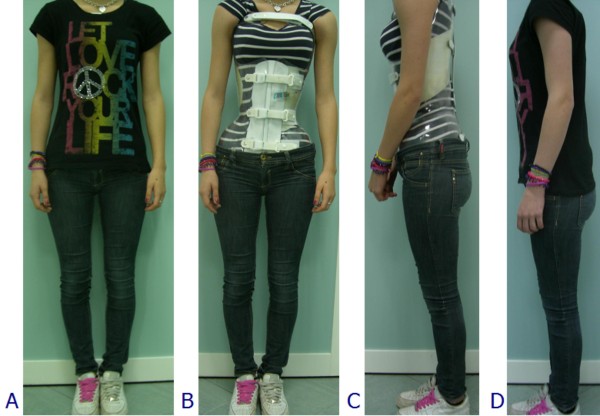
**The Sforzesco brace invisibility**. The Sforzesco brace was developed in a town of fashion (Milan), and some patients have stated that this is reflected in its design, that increases wearability.

**Figure 7:**
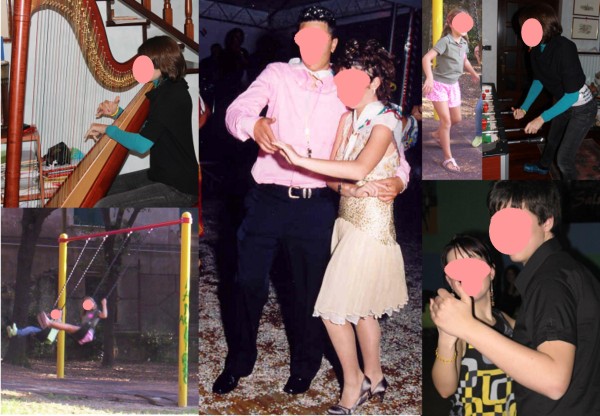
**Patients want correction and an invisible brace**. These patients are wearing their braces in various everyday activities.

From a theoretical perspective, the authors started this research with very well-established principles of correction that had developed over the years. These principles are divided in terms of efficacy (type and quality of the brace) and acceptability (compliance). The efficacy principles include [31,32]: mechanical efficacy, the active brace principle http://www.youtube.com/watch?v=u87UonO-1Yg&feature=player_embedded, versatility and adaptability, teamwork, compliance. The acceptability principles of correction (meaning compliance as well as a human approach to the patient) include: perfect body design and minimal visibility (Figure 6), maximum freedom in the Activities of Daily Life (Figure 7), assumption of responsibility, cognitive-behavioural approach by the entire professional team [33].

The SPoRT acronym [10,17,18,31,34], developed according to these principles, means: Symmetric, Patient-oriented [35], Rigid, Three-dimensional, active.

## The Brace

Three braces follow the SPoRT concept of correction. The Sforzesco brace (Figure 1) is constructed with rigid polycarbonate, in two pieces, connected posteriorly at the midline by a vertical aluminium bar and anteriorly by a closure that is rigid over the breast and below is made of soft inelastic bands. While the brace appears to be in full contact, in reality due to its symmetry and according to the theoretical body shape the patient would have without scoliosis, it provides space over depressions and pushes over pathological elevations.

**Figure 8:**
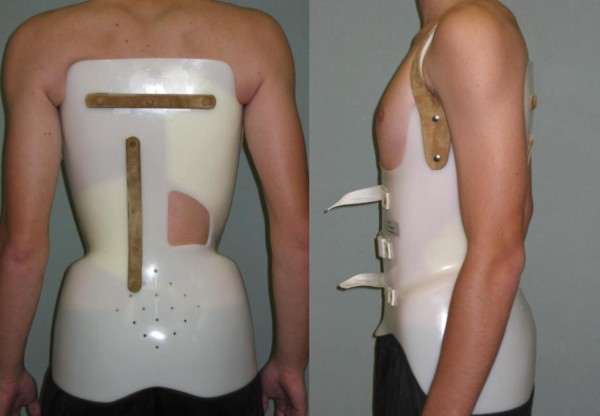
**The Lapadula brace to treat scoliosis and hyperkyphosis**. The Lapadula brace has much versatility and can be adapted to treat an hyperkyphosis associated with scoliosis.

The other two braces are made of polyethylene. In terms of construction and correction approach, the Sibilla (Figure 2) and Lapadula braces (Figure 3) are completely analogous to the Sforzesco brace, and therefore they will be considered together. The only difference between the two is that the Lapadula brace does not have the upper plastic part over the breast (it also addresses kyphosis in combination with scoliosis through the use of acromion metallic pushes - Figure 8).

**Figure 9:**
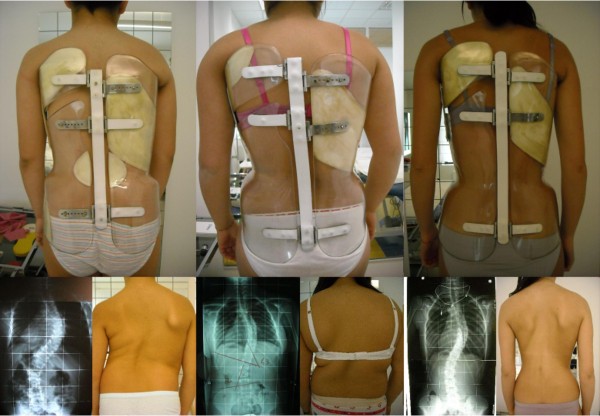
**The first Sforzesco brace causes a sudden lengthening of the trunk requiring correction in 2 months**. Typical correction made to the first Sforzesco brace after a wearing period of 2 months in patients with important thoracic curves: it becomes too short under the concave shoulder and must be lengthen.

The main innovation of the SPoRT braces can be found in the concept of drivers. This was introduced for the first time in bracing with the Sforzesco brace [10,31], and was discovered due to the abundance of material used to guarantee the rigidity that was necessary to emulate the strength of the Risser cast. This material no longer allowed the trunk to escape from the pushes: the only real escape remaining to the spine as soon as the maximum external symmetry is achieved (i.e. the drivers are reached) is in elevation (Figure 9).

Correction is reached through construction (shape of the envelope), pushes, drivers (concept newly-introduced with this brace), escapes, stops.

## Practical Issues

### How to prescribe the brace and principles of correction

Prescribing the SPoRT braces requires a careful three-dimensional evaluation of the characteristics of the curve of each single patient. Clinical reasoning follows a systematic path by looking progressively at the single component of the deformity.

### Frontal plane correction

#### The slopes

**Figure 10:**
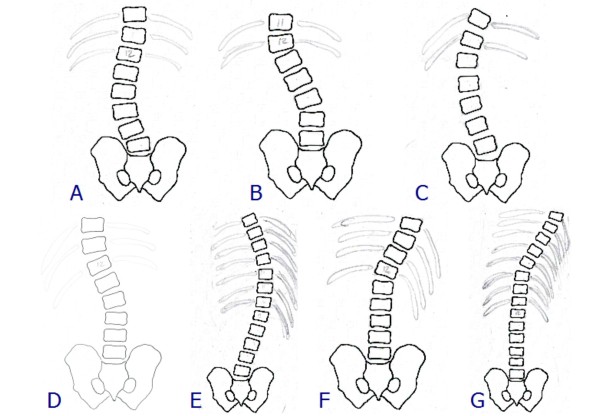
**The slopes**. The slopes. Low lumbar (A); High lumbar (B); Lumbar (C); Thoracolumbar (D); Thoracic (E); Distal thoracic (F); Proximal thoracic (G).

Correction on the frontal plane is based on the identification not of the curves (as usual), but of the slopes, that are the most frontally flexed segments of the spine. In fact, since the brace works by pushing the spine from below, and due to the presence of the drivers that avoid undesired actions, pushes are focussed on the most severely flexed area of the spine (slopes). In a down-up direction, the following slopes can be described:

• Low lumbar (Figure 10A): in a lumbar curve, below the apical vertebra.

• High lumbar (Figure 10B): in a short thoracolumbar curve, below the apical vertebra; or in a very short lumbar curve, above the apical vertebra.

• Lumbar (Figure 10C): in a wide thoracolumbar curve, below the apical vertebra.

• Thoracolumbar (Figure 10D): in a lumbar curve, above the apical vertebra; or in a low thoracic curve, below the apical vertebra.

• Thoracic (Figure 10E): in a thoracolumbar curve, above the apical vertebra; or in a single thoracic curve, below the apical vertebra; or in a double thoracic curve, above the apical vertebra of the distal curve and below the apical vertebra of the proximal one.

• Distal thoracic (Figure 10F): specified only in Double Moe curves where three thoracic slopes are present, below the apical vertebra of the distal curve.

• Proximal thoracic (Figure 10G): in a thoracic curve, above the apical vertebra.

**Figure 11:**
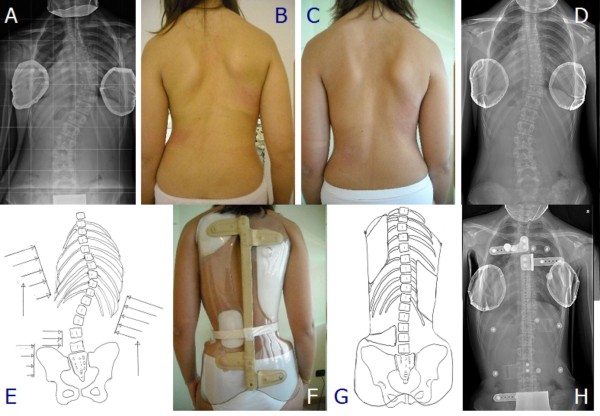
**Example of correction on the frontal plane slopes**. Example of correction on the frontal plane slopes of one patient with a thoracic curve of 48° (Risser 1). (A) X-ray pre-brace (48°); (B) aesthetics pre-brace; (C) aesthetics after 4 months of bracing; (D) x-ray without the brace after 4 months of bracing: corrected to 23° (i.e. reduction of 25°, -52%); (E) pre-brace planning with pushes on the right thoraco-lumbar and left thoracic slopes, as well as a stop to the right of the flanks; (F) constructed brace; (G) application of the pushes in the constructed brace; (H) in-brace x-ray with a correction to 13° (i.e. 35° of correction, 73%).

When evaluating slopes, it is important to decide which is the most important to correct and where the orthotist (CPO) should focus in constructing the brace. Once the main slopes to be corrected have been defined, the correction follows automatically as shown in an example in Figure 11. In Table 1 the corrections to be made according to the identified slopes are reported.

**Table 1:** Corrective action according to the frontal plane identified slope

	Slope	Action	Construction
1	Low lumbar	shift of the trunk at the base	The whole trunk is shifted toward the concavity of the lumbar curve

2	High lumbar	elevation of the emithorax	The last ribs on the side of the convexity are elevated with a gradually decreasing compression in a down-up direction

3	Lumbar	shift of the trunk at the base and elevation of the emithorax	Combination of 1 and 2, on the same side

4	Thoracic	push on the distal ribs below the apical vertebra	On the side of convexity of the curve. All the ribs involved in the slope must be pushed. The rib corresponding to the apical vertebra is not involved (and sometimes also that below the apical)

5	Distal thoracic	push on the distal ribs above the apical vertebra	On the side of concavity of the curve

6	Proximal thoracic	push on the distal ribs above the apical vertebra	On the side of concavity of the curve

7	Thoracolumbar	elevation of the emithorax and push on the distal ribs below the apical vertebra	Combination of 2 and 4, on the same side

At the thoracic level, the ribs to be pushed must be identified, corresponding to the flexed vertebrae avoiding the apical vertebra.

The possible actions (not mutually exclusive) at the flanks include:

• Shift: in the case of a low lumbar slope.

• Stop: when there is a lumbar curve on the side opposite to the main slope.

• Remodelling: to improve the aesthetics of one flattened flank.

**Figure 12:**
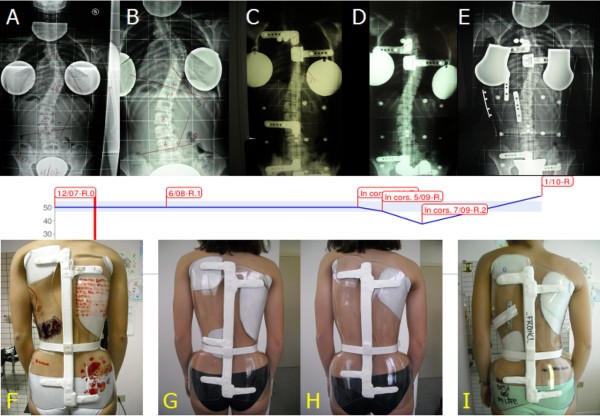
**The Cheneauisation of the Sforzesco brace**. We used the term Cheneauisation to underline that it derives from the contamination of our own brace with the internationally well-known Cheneau brace: in fact it aims at posturally changing the scoliosis curve through a thrust at level of the convexity of the apical proximal curve and an elevation/medialization of the shoulder at the concave side. Due to our own SPoRT principles the Cheneuization also includes an anteposition of this same shoulder. First line, from left to right: (A) x-rays of the patient at start of treatment (12/07), (B) after 6 months (6/08), (C) in-brace without Cheneuization (4/09); (D) in-brace with Cheneuization (5/09), (E) in-brace with Cheneuization after two months of treatment (7/09). Second line: graph of x-rays measurements. Third line, from left to right: (F) the first brace used; the brace trial: (G) without Cheneuization; (H) with Cheneuization; (I) the brace with Cheneuization at time of the 7/09 x-ray. This is the first situation in which we used the Cheneuization due to the absence of correction in a patient with an high-degree scoliosis refusing surgery, and presenting with a curve possibly responding to such a change. We then made two braces and compared their results with in-brace x-rays, with favorable results for the Chenuization (5/09), that was even increase by time and brace corrections (7/09). The final out-of-brace progression of scoliosis (1/10) was due to a sudden decrease on brace usage that this specific patient suffered.

**Figure 13:**
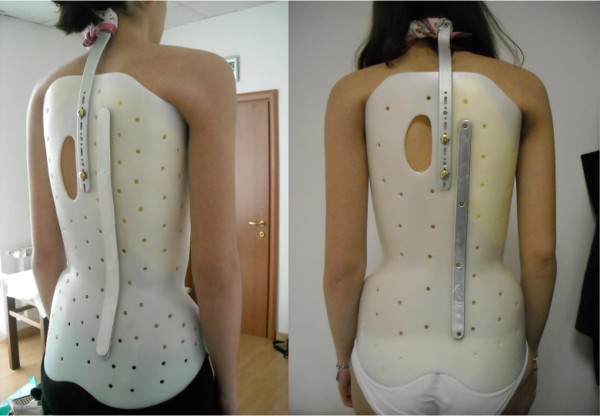
**Cervical push on the transverse process of C7 and above**. Cervical push on the transverse process of C7 and above.

One main point to be carefully considered is the correction of high thoracic slopes above the T5 vertebra. Over the years, many possible solutions have been tried, including pushes on the cervical transverse processes, elevation of one shoulder, and finally something called "Cheneauisation", that is an inclination of the entire brace above the apical vertebra of the thoracic curve opposite to the proximal slope, together with an advancement of the shoulder on the same side (Figure 12). The term Cheneauisation was used to underline the fact that it derives from the contamination of our own brace with one of the other most well-known braces at the international level, the Cheneau brace. A cervical push on the transverse process (Figure 13) can be prescribed in many situations when it is deemed important to act on the cervico-thoracic junction.

#### The drivers

On the frontal plane, the main drivers are placed laterally on the concave side, i.e. at the level of the waist and/or the thorax. They act mainly in a down-up direction from the apical vertebra of the curve, even if their action starts where the contra lateral push begins. They direct the forces above.

### The horizontal plane

**Figure 14:**
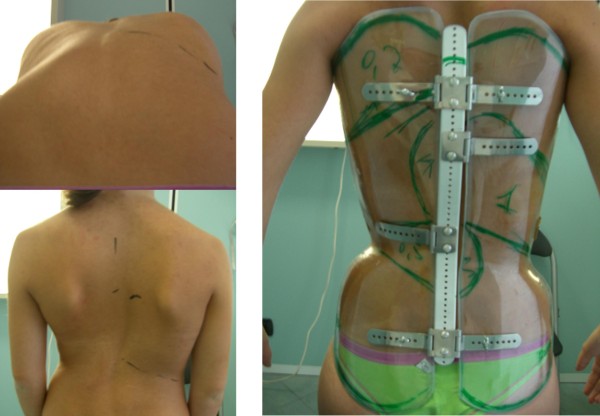
**Identification of the prominence to localize the derotation pushes**. Identification of the prominence to add plastazote pushes to the envelope. On the left, top down direction: anterior bending to precisely identify the hump height and ribs involved and mark them; markers on the skin in standing. On the right: marks reported on the brace.

The correction on the horizontal plane is totally based on the hump characteristics combined with the needs on the sagittal plane. In general the push is realised with a plastazote addition inside the external envelope following exactly the apparent prominence, as shown in Figure 14.

**Figure 15:**
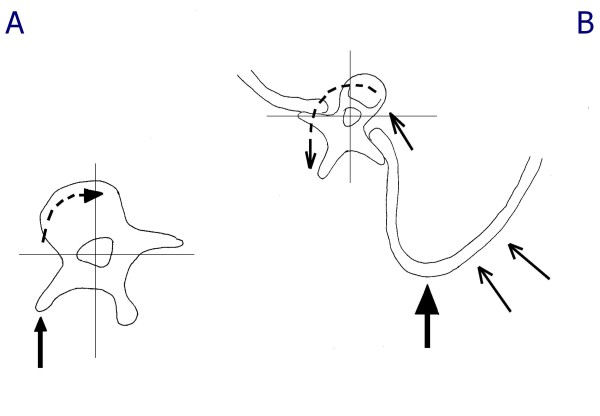
**Action of the derotation pushes at thoracic and lumbar levels**. At the lumbar level, the push on the hump helps to reconstruct lordosis, and as a consequence it is directly on the transverse apophysis, potentially also adding a frontal plane corrective action (A). At the thoracic level, on the contrary, the push can damage the sagittal plane, and must not reach the transverse processes, so to allow a possible leverage by the rib that could even result in a kyphotisating action (B).

At the lumbar level, any horizontal derotatory push on the hump corresponds to a useful reconstruction of the lordosis usually needed in this area. There are no real concerns of sagittal damage. As a consequence, the push is directly on the transverse apophysis, which can potentially also add a frontal plane corrective action (Figure 15A). Obviously, in the rare cases of associated hyperlordosis all the brace will be built in delordosis.

At the thoracic level, on the contrary, the derotatory push can damage the sagittal plane, and must be carefully planned. In this respect, it is mandatory not to reach the transverse processes, so as to allow for possible leverage by the ribs that could even result in a kyphosing action (Figure 15B). This leverage is at the base of the derotation and possibly deflexion action of the push on the hump. Moreover, the push must be below the apex of kyphosis to avoid its flattening. Above it, the push should be on the proximal counter-rotation appearing as a consequence of the thoracic thrust on the hump. This will allow on one hand an action to reconstruct the kyphosis, and on the other hand will increase the direct derotating (and modelling) push on the hump, as well as a realignment of the shoulder girdle otherwise rotated opposite to the convex side of the curve, due to the push on the thoracic hump.

At the thoracolumbar level, the action is usually similar to that at the lumbar one. In fact, most of the cases in this region appear with a junctional kyphosis, which is contrasted by a posterior push on the hump. In the few cases in which a junctional lordosis is present, the push must be present, but moderate to avoid increasing the sagittal deformity.

#### The drivers

On the horizontal plane, the main drivers are anterior, where they avoid the anterior escape of the trunk driving in rotation, and posterior on the opposite side of the push, which are reached only when complete derotation is achieved and the push is driven upward.

### The sagittal plane

This correction is almost completely done through the construction, since afterwards during checks it is almost impossible to really correct this point. The sagittal shaping of the brace during construction almost always changes according to the given patient's sagittal curve.

#### The drivers

On the sagittal plane, all the drivers previously listed for the other planes play a crucial role in driving the forces not only upward but also slightly backward at the thoracolumbar junction, and anteriorly over the apex of kyphosis.

### How to build the brace

The SPoRT concept always requires a customised construction of the brace according to the patient's individual requirements. CAD-CAM technologies usually allow us to obtain the best results, without using pre-built forms stored in databases, as is often done by others. Orthotists must directly shape the scanned trunk according to the patient's requirements. Once done, a final test must be made on the patient so as to change the first theoretical project and adapt it in the best possible way, depending on the real interaction between the body and the brace.

**Figure 16:**
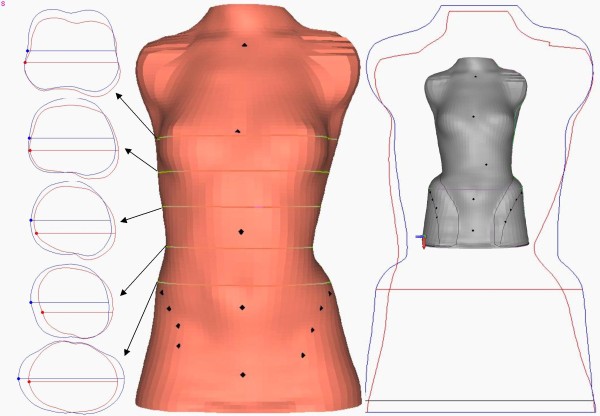
**Modelling of the trunk shape during brace building**. During brace building a careful modelling of the trunk shape is made either on the cast mould or on the PC screen. In this figure the correction of circumferences (left) and shapes (right) of a single patient with a right thoraco-lumbar curve is reported. On the left there are the original (red) and corrected (blue) horizontal sections of the body at the level of the horizontal lines reported in the middle body shape, where the original laser scan of the trunk is represented. On the right the frontal contour of the original (red) and corrected (blue) body shapes are reported, while inside these lines, in grey, there is the final trunk shape from which the brace is going to be built.

The brace is built through careful modelling of the trunk shape either on the cast mould or on the PC screen. The cast is sometimes constructed in a step by step procedure in down-up direction already trying to achieve a good correction. At first, maximum symmetry is searched for among the trunk volumes in three dimensions, looking at circumferences (Figure 16A) and shapes (Figure 16B). Then, the sagittal plane is shaped. Finally, all planes are re-checked.

When the mould is ready, the plastic TLSO is fabricated, and the patient is fit according to his/her needs, allowing for good sitting position and total freedom of movement. The pushes are finally added at the level of the humps according to the desired corrections.

### How to check the brace

Brace checking is a fundamental step in any brace construction [4]. This is especially true in braces following the SPoRT concept, since they are adjustable and customisable according to any individual curve pattern. The reaction of the body to predisposed project of the brace should also be considered during prescription and building. Brace checking is moreover a key psychological intervention on the patient and family, mainly, but not only, with the first brace.

On the frontal plane, one has to search for the area in which correction is not ideal: corrections may be applied increasing the pushes or decreasing the drivers and counter pushes. On the sagittal plane, besides the appearance of the brace that must be properly aligned with respect to a normal kyphosis and lordosis, it is necessary to check inside the brace, and eventually either act on the posterior aluminium bar of the brace, or add plastazote pushes. On the horizontal plane, the check is made without the brace looking at the effect of the pushes on the humps. Finally, the total balance of the braced trunk is assessed, to avoid sagittal or frontal shifts (and rarely horizontal rotations). Other technical points to be checked include the non-overlap of pushes, that must be done on a plane by plane basis and the balance among the pushes (in the Sforzesco brace, pushing too much on a secondary curve has the consequence of reducing the efficacy on the main one).

An "in brace" radiograph is usually done only once, almost 45 days after the initial fitting of the first brace, but sometimes more often if there are problems.

### Protocols and everyday usage

Brace treatment must almost always achieve very good aesthetic body shaping [36]. It is intended to achieve radiographic results that are compatible with good functioning of the spine in adulthood, while the quality-of-life impact and psychological disturbances due to the brace must be minimised [5,17,37].

The type of brace is chosen according to the rigidity of the scoliosis to be treated. In large curves (over 40°), that are always rigid, the Sforzesco brace is used. Before puberty, in juveniles or infantile scoliosis patients, the Sibilla brace is prescribed with the very rare exceptions of a very rigid curve; in all other clinical situations, a case by case choice is made. The Lapadula brace is used as an alternative to the Sibilla in lumbar and thoraco-lumbar curves.

The goal of brace treatment varies according to the degree of curvature considered, and the forces that(in terms of rigidity of the brace and the hours of usage) are consequently administered [31]. Treatment is tailored according to individual preferences, anthropometric characteristics and other risk factors such as rotation, hump, lumbar curve take-off, imbalance, etc. It usually starts at full time. Actually, the applied full-time concept varies between 18 and 23 hours per day [3,38] with the goal of obtaining compliance. Treatment is carried out by wearing the brace at least 18 hours per day until the period of rapid growth is over and other adjustments due to the pathology are not foreseen. This is usually achieved at Risser stage 3.

**Figure 17:**
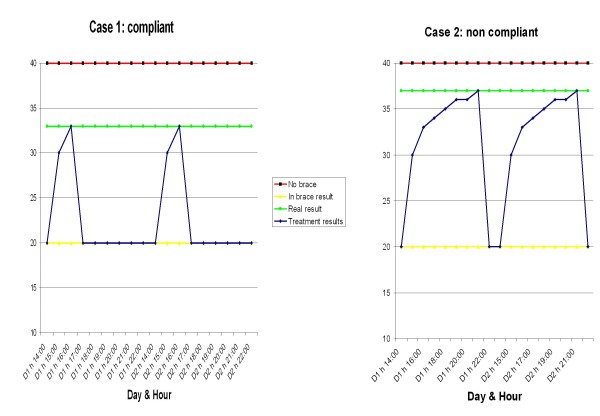
**The concertina effect of brace correction**. The concertina effect [31] could explain the importance of patient's compliance. According to this hypothesis, each time a brace is weaned the deformity gradually moves back from the maximal in-brace correction to the original out-of-brace situation; this reversal is due to a postural collapse [39-41], that is correlated to the length of brace weaning and the rigidity (flexibility) of the spine [39] (itself correlated to the stage of growth, the bone age, the muscular endurance and the usual brace wearing). According to the "concertina effect" hypothesis, the deformity reached at the end of daily brace weaning gives the allowed compression of the wedged vertebrae, and consequently the final results. In fact, the more the brace is weaned daily, the worst the results. We published some preliminary proves of this hypothesis [71].

Weaning requires a two-hour reduction every six months. This protocol has been developed in our Institute over many years in order to help the postural neuro-muscular system maintain the achieved correction [31] as well as to maximize compliance. In fact, while scoliosis is a bone deformity, there is also a postural component of the curve [39] that always increases it [40] and can be the basis of its progression [31,41]. Moreover, while movement has been shown to be a crucial progression factor [42,43], it can also be reorganized to become a stability factor [44]. Braces directly interfere with such neuromuscular functions [1,2,41]. Because posture and movement require long-term adaptations [41,45], the longer the weaning phase, the better the neuromuscular system should adapt, hopefully maintaining the inputs received by the brace even after complete weaning. In this respect, proper stabilization exercises should play a major role reducing the concertina effect (Figure 17) [31,46]. All this should positively interfere with bone tissue formation [42,43], even if the postural system *per se *is part of the problem to be corrected [39-41].

### Exercises

**Figure 18:**
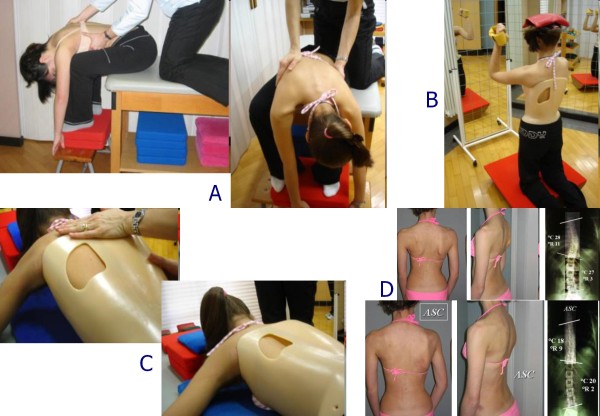
**Examples of SEAS exercises to be performed during brace treatment**. SEAS exercises during brace treatment. (A) Preparation to bracing. Exercises aimed at increasing range of motion of the spine on all planes, in order to allow the brace to exert the maximum possible correction. (B) Modeling exercises in brace. The patient is in a relaxed position The patient moves away from sternal upright to do a maximum thoracic kyphotization movement. (C) Muscular endurance strengthening exercises. We propose strengthening exercises, requiring lumbar lordosis and thoracic kyphosis preservation, while frontal and cross-sectional plans correction is guaranteed by brace pushes. (D) Active Self-Correction (ASC) (autocorrection according to SEAS) during brace weaning.

We apply the SEAS (Scientific Exercises Approach to Scoliosis) exercises as developed by ISICO in these years [31]. The main goals of exercises in brace treatment are elimination or reduction of side effects caused by immobility (muscular hypotrophy and joint rigidity), or the brace itself (reduction of sagittal curves, mainly kyphosis, and breathing impairment) and accentuation of brace corrective pushes [47-49]. Moreover, exercises aim at not loosing correction while weaning the brace [46]. Such goals are pursued through specific therapeutic modalities, subdivided into the following three treatment phases: preparation for bracing (Figure 18A); brace wearing period (Figure 18B and 18C); complete brace weaning (Figure 18D) [31].

We have recently shown in prospective controlled studies the importance of exercises in preparation for brace treatment so to increase its efficacy at first wearing [50], and in retrospective studies the usefulness of SEAS exercises in order to not lose correction while weaning from the brace [46]. We have also shown which exercises are more useful in increasing the pushes of the brace [49].

## Results and case reports

**Figure 19:**
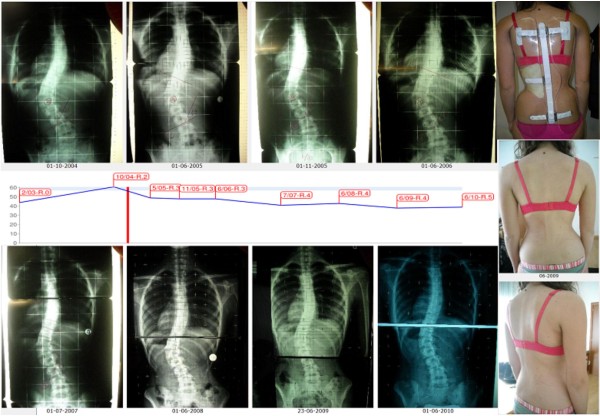
**First case report: adolescent thoraco-lumbar scoliosis patient over 45° who reached the end of treatment**. On the left, from left to right, in the first and then in the third line: all x-rays of this case report of a patient that reached the end of treatment (each x-ray is marked with the corresponding date). On the left, midline: graph with the results obtained, dates and Risser test. On the right, in top-down direction: the brace used, and the posterior and sagittal aesthetic appearance at the end of treatment. A.A. has been evaluated the first time in December 2004, presenting with a second x-ray showing a thoraco-lumbar left scoliosis progressed in 18 months from 44° (Risser 0) to 61° (Risser 2). Fusion had been proposed, but refused. She started treatment with the Sforzesco brace 23 hours per day and SEAS exercises 3 times a week (45'): after 5 months she was 49° (Risser 3). Treatment continued other 6 months 23 hours per day, then 22 per 6 months, and brace was continously and gradually weaned 2 hours every 6 months: she improved after 3 years of treatment (41°, Risser 4) and 4 years (38°, Risser 4). At the last x-ray after 48 hours without the brace, and 5 years and 6 months of continuos Sforzesco brace treatment and SEAS exercises, she finished treatment at 39°.

**Figure 20:**
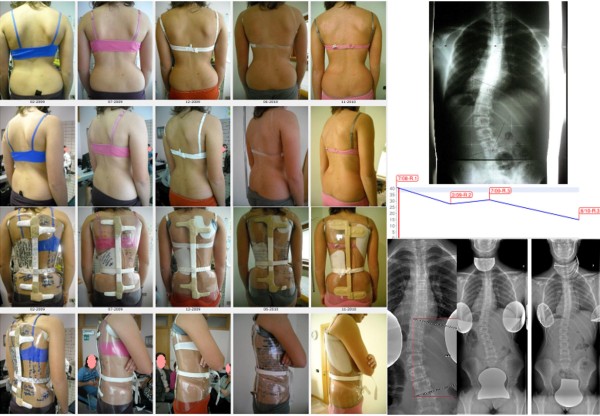
**Second case report: adolescent thoraco-lumbar scoliosis patient still in treatment**. On the left: posterior (first line), and sagittal (second line) aesthetics, and the brace in use (posterior - third line; lateral - fourth line) of all evaluations (apart the first visit) are reported from left (oldest) to right (last one). On the right, top-down, left-right direction: all x-rays of this case report of a patient still in treatment, and the graph with obtained results. C.S. has been evaluated the first time in July 2007, presenting with the first x-ray showing a thoraco-lumbar left scoliosis of 41° (Risser 1): fusion had been proposed but refused. She started treatment with the Sforzesco brace 23 hours per day and SEAS exercises 3 times a week (45' per session): after 6 months she was 28° (Risser 2). Treatment continued 6 months 22 hours per day, then with a 2 hours progressive weaning every 6 months. At the last x-rays after 2 years of treatment, performed after 8 hours without the brace, she was improved to 15° (Risser 3). Now she is wearing the brace 14 hours per day.

**Figure 21:**
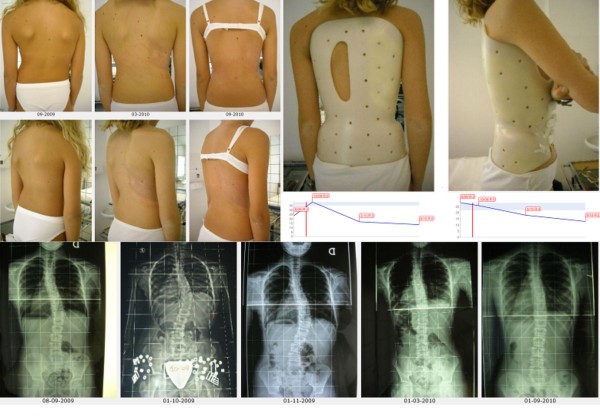
**Third case report: adolescent thoraco-lumbar scoliosis patient rapidly progressing still in treatment**. On the left: posterior (first line), and sagittal (second line) aesthetics. On the right: the brace used, and the graph with obtained results. On the bottom line: all x-rays of this case report of a patient still in treatment. G.B. presented in september 2009, 10 years old, with a first x-ray showing a thoracic left, thoraco-lumbar right curve of 28°-24° (Risser 0): parents stated that they had seen their daugther worsening in the 15 days span between the x-ray and the medical evaluation. At first a SpineCor brace has been prescribed but the x-ray within brace showed such a bad situation (14°-30°) to suggest to re-evaluate a radiograph without the brace: scoliosis was rapidly worsening (26°-39°). We decided to move to a SPoRT brace and SEAS exercises (twice a week, 45' per session): Sibilla 23 hours per day. In 6 months, while growing 6 cm. (from 145 to 151), she corrected to 17°-18°, and in 6 more months wearing the brace 22 hurs per day, she arrived to 13°-14°, during an height increase of other 6 cm. (from 151 to 157). Now she continues to be Risser 0, and is wearing the brace 21 hours per day.

**Figure 22:**
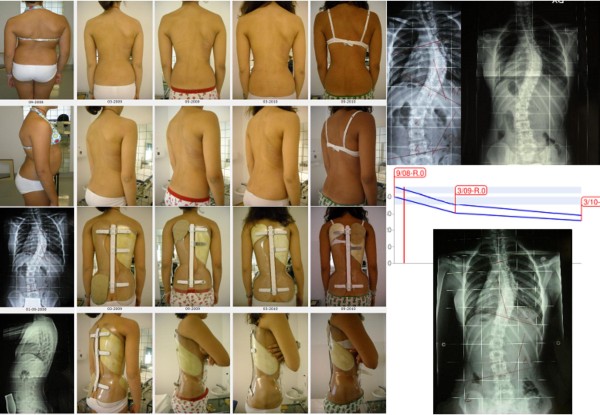
**Fourth case report: adolescent double thoracic, lumbar scoliosis patient over 45° still in treatment**. On the left: posterior (first line), and sagittal (second line) aesthetics, and the brace in use (posterior - third line; lateral - fourth line) of all evaluations (apart the first visit, where aesthetics and first x-rays are shown) are reported from left (oldest) to right (last one). On the right, top-down, left-right direction: all x-rays of this case report of a patient still in treatment, and the graph with obtained results in the two curves: upper line, thoracic curve, lower one lumbar curve. C.F. has been evaluated the first time in September 2008, presenting with the first x-ray showing a thoracic right lumbar left scoliosis of 46°-39° (Risser 0). She started treatment with the Sforzesco brace 23 hours per day and SEAS exercises 2-3 times a week (45' per session): after 6 months, while growing 5.5 cm. (from 158.5 to 164), she was 36°-31° (Risser 0). Treatment continued 6 more months 23 hours per day, then reduced to 22: in this year, while growing 4 cm. (to 168), she reduced her scoliosis to 29°-27°. After 6 months at 20 hours, now she is wearing the brace 18 hours per day.

**Figure 23:**
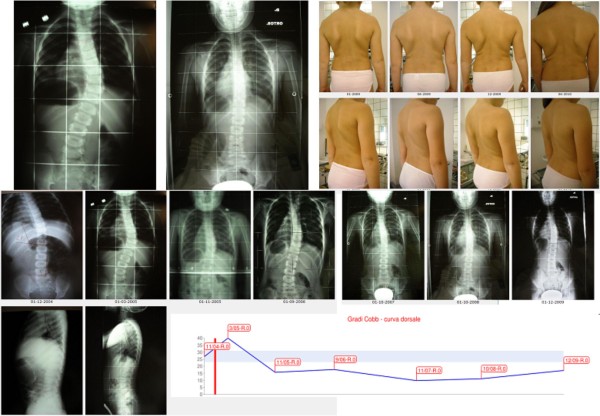
**Fifth case report: infantile thoracic scoliosis patient of 45° who weaned the brace but is still in treatment**. First line, left to right: x-rays at brace wearing (March 2005) and at brace weaning (October 2008), frontal and sagittal aesthetics in the last evaluations after brace weaning. Second line: all frontal x-rays of this case report of a patient still in treatment. Third line: sagittal x-rays, and the graph with obtained results. B.C. infantile thoracic right scoliosis was discovered at the age of 13 months, and rapidly progressed without treatment from 27° to 40° in 4 months as soon as she reached the standing position. A Sibilla brace treatment was then started 23 hours per day per 4 months and 20 during the summer, with an immediate reduction to 16° in 8 months. Brace was then gradually reduced 2 hours every six months while maintaining correction, and finally weaned with the curve at 10°. At the age of 6, as soon as was possible, everyday SEAS exercises (20' per session) have been started and are the only actual treatment.

The short term results currently available on the SPoRT concept relate to the Sforzesco brace and are quite promising. Although the first treated patients have already reached the end of treatment, there are not yet enough of them to be able to perform a formal study. Nevertheless, even if we are perfectly aware that clinical case reports (Figure 19, 20, 21, 22, and 23) are not comparable to strong scientific data coming from other studies, those we presents here convey in our view an important message to the reader and allow a deeper understanding of the effectiveness of this brace.

With specific studies we have shown that the Sforzesco brace:

• is more effective than the Lyon brace after six months of treatment, with a matched case/control prospective study [10,18] on 30 AIS patients aged 13 years and with curves of 38° Cobb on average: in the Sforzesco group 80% of patients improved and none worsened, while the Lyon group had results of 53% and 13%, respectively.

• is equally effective as the Risser plaster cast to achieve the maximal correction after 18 months of treatment, with a prospective cohort study with a retrospective control group [34] on 41 AIS patients aged 14 years and with curves of 40° Cobb. The Sforzesco brace was shown to be more effective at reducing the thoracic curve, and its results were super imposable for the other regions. The Risser plaster brace was shown to be more effective on the thoracic hump and in regard to the cosmetic appearance of the flanks, but it also caused a serious reduction in kyphosis.

• is more effective than the Risser cast + Lyon brace in treating curves over 45° Cobb at the end of growth, with a prospective cohort study in patients who utterly refused surgery [51] on 28 AIS patients aged 14 years and with curves of in a range of 45° to 58° Cobb. The patients braced with the Sforzesco had better results than those treated with the Risser cast in the thoracic curves, without any sagittal plane worsening. For the other parameters, the results were similar.

• is able to improve aesthetics in scoliosis patients, with a prospective not-controlled cohort study [52] on 34 consecutive AIS patients 13 years old with curves of 32° Cobb with Aesthetic Index (AI) [36] scores of at least 5/6. At baseline, median AI was 6 (95% IC 5-6), but the score decreased to 3 (95% IC 0-5; p < 0.05) after six months with the brace, and this value was maintained in the 29 who completed the treatment (95% IC 1-6; p < 0.05 with respect to baseline).

## Discussion

The Sforzesco brace has been developed recently, but it is already one of the most tested TLSOs in the very weak scientific history of bracing. We are not able to compare it with any other that we did not use personally, but we can already state according to our results that its efficacy is higher than that of the Lyon brace [10], and comparable (or even higher as well) to that of the Risser cast [16]. In fact, we use to think of the Sforzesco brace as a cast, with the great advantage on one hand that it can be removed to greatly increase patient comfort, and on the other hand that it can be used from the beginning to the end of treatment without problems, which cannot be done with the Risser cast. We cannot exclude in the future the possibility that the cast (or the Lyon brace) will still find a place in scoliosis treatment for some particular curves or patients, but we are not able now to exactly identify these clinical situations.

According to the reported results, we have a strong basis for reasoning that this brace could be more effective in the worst curves than other braces. In fact, to our knowledge, there is only one published paper with good results on curves over 45° Cobb, and they have been obtained either with Risser casting or with Sforzesco bracing [51]. This conclusion needs to be supported by future evaluations and understanding, as well as study results reported by others with other braces.

Limitations can obviously be found today in the fact that the use of this brace is limited to Italy; we can anyway already state that the usage of the Sforzesco brace has already spread outside the first orthotic manufacturer and the first physician and his team. Nevertheless, we need studies from other teams, as is common with instruments at their first stages of development. A typical disadvantage of this instrument is that it is apparently simple. In fact, to a superficial observer it could appear as a simple full-contact brace. In reality, there are complex mechanical concepts and understanding that must be developed to be able to correctly apply this family of braces. Its apparent simplicity could easily drive its spread but could also lead to misconduct in its application. Moreover, another disadvantage is that the messages given to the patients are vital to success as well, and must be well understood. The SPoRT concept could also be applied to other braces beyond the ones presented here.

## Conclusions

Looking at the braces used around the world, most of them are based on three-points systems, more or less three dimensional [26,28,53-61], but we can also recognise a traction system [62-64], a postural one [65-67], and finally a corrective-movement based [44,68]. The SPoRT concept of bracing, due to its three-dimensional action of elongation pushing the spine in a down-up direction, is different from all the other corrective systems. The Sforzesco brace appears as the best brace for the worst curve magnitudes, being comparable to casting [16,51,69], with the obvious advantage of being removable and applicable for all duration of treatment.

Bracing is very hard work, in terms of conceptualisation of the practical work to be done, and of the interaction with the whole team, starting from the physician-orthotist relationship, to the physiotherapist, the patient and the family. It is a demanding, progressive, slow, artisanal effort in the art of patience. In this respect, it is quite the opposite of the short, one-shot, quick, highly demanding, current surgical fusion. As we use to say to our patients, bracing corresponds to the very slow pace of building oneself that humans usually have to face, contrary to the fast solution that he/she may tend to prefer and see as less demanding. Bracing in this respect also becomes a philosophy of one's approach to life, and this is one reason why it is difficult that the slow pace of a good conservative physician can also be the fast speed of a good orthopaedic surgeon, and *vice versa*. As well, there will always be patients who prefer bracing and others who prefer surgery. This relates to those with high degree curves; in low degree curves, the choice is between bracing and a "wait and see" strategy, applied in cases in which bracing is too demanding for that particular patient. But, in our own experience, at least in Italy, this is very rare [70], even if not avoidable.

## Competing interests

SN is a physician everyday prescribing braces; he also owns a stock of ISICO.

GM is an Orthotist everyday building braces and owns Centro Ortopedico Lombardo.

FT is an Orthotist everyday building braces and owns Orthotecnica.

## Authors' contributions

SN drafted the text and figures. GM and FT revised and accepted it, and contributed with some figures. All authors read and accepted the final version of the manuscript.
